# Medicare Parity and Outpatient Mental Health Service Use and Costs Among Beneficiaries With Depression

**DOI:** 10.1001/jamanetworkopen.2025.8491

**Published:** 2025-05-02

**Authors:** Sonia M. Tetlow, Victoria L. Phillips, Jason M. Hockenberry

**Affiliations:** 1 Department of Health Policy and Management, Emory University, Atlanta, Georgia; 2 Department of Health Policy and Management, Yale School of Public Health, New Haven, Connecticut; 3 Now with Global Health Center, Centers for Disease Control and Prevention, Atlanta, Georgia

## Abstract

**Importance:**

Less than half of the US population with any mental health condition receives services. Cost is the most commonly cited barrier to treatment.

**Objective:**

To examine whether service use and out-of-pocket expenditures among Medicare beneficiaries with depression changed after Medicare implemented equal cost-sharing for outpatient mental health and medical services (Medicare parity).

**Design, Setting, and Participants:**

This economic evaluation used a single-group, interrupted time series design and examined data from the Medical Expenditure Panel Survey Household Component from 2008 to 2019. The sample included Medicare beneficiaries aged 65 years or older with depression. Data were analyzed from June 2, 2023, to June 17, 2024.

**Exposure:**

Under the Medicare Improvements for Patients and Providers Act of 2008, beneficiary cost-sharing for outpatient mental health services decreased from 50% prior to 2010 to 20% in 2014, creating parity with equivalent medical care.

**Main Outcomes and Measures:**

The primary outcomes were outpatient mental health service use, as assessed by mean use, proportion of beneficiaries with any use, and intensity of use (ie, mean use among users), and out-of-pocket expenditures.

**Results:**

The analysis included 5831 Medicare beneficiaries. Using the Medical Expenditure Panel Survey person-level survey weights, this number corresponded to a nationally representative sample of 72 436 656 beneficiaries (median [IQR] age, 72 [68-79] years; 64.2%-72.2% female per study year). After Medicare parity, mean use of outpatient mental health services among beneficiaries with depression increased by 0.54 visits per year (95% CI, 0.31-0.76 visits per year), and proportion of use increased by 6.61% per year (95% CI, 2.23%-10.99% per year). Intensity of use decreased at parity by a factor of 0.90 (95% CI, 0.82-1.00) and increased after parity by a multiple of 1.07 per year (95% CI, 1.04-1.10 per year). Mean out-of-pocket expenditures for these services increased after parity by $12.25 per year (95% CI, $2.42-$22.08 per year). Sensitivity analysis using the 2016 US Preventive Services Task Force recommendation for routine adult depression screening indicated that the proportion of use increased 28.26% (95% CI, 24.33%-32.19%) once the recommendation was issued.

**Conclusions and Relevance:**

In this economic evaluation of Medicare parity, implementation of Medicare parity coupled with routine adult depression screening was associated with significant increases in outpatient mental health service use among Medicare beneficiaries with depression. These findings suggest that parity policies alone may not be sufficient to effectively address multiple barriers to mental health care but in tandem with physician screening, diagnosis, and referral practices, may increase the accessibility of mental health services.

## Key Points

**Question:**

Did outpatient mental health service use and out-of-pocket expenditures change among Medicare beneficiaries with depression after cost-sharing parity?

**Findings:**

This economic evaluation of 5831 beneficiaries found that parity was associated with increases in mean use of outpatient mental health visits (0.54 visits per year), use among users (by a multiple of 1.07 per year), and mean out-of-pocket costs ($12.25 per year). Sensitivity analyses indicated that policy recommendations around routine depression screening following parity also influenced service use.

**Meaning:**

These findings suggest that complementary policies that reduced cost barriers and encouraged depression screening and treatment referrals may have contributed to increased mental health service use among Medicare beneficiaries with depression.

## Teaser

This economic evaluation examines whether service use and out-of-pocket expenditures among Medicare beneficiaries with depression changed after Medicare implemented equal cost-sharing for outpatient mental health services.

## Introduction

Mental health conditions are common among US populations, including older adults (aged ≥50 years), yet less than half of people with any mental health condition on average receive mental health services.^1,2^ Cost is one of the most commonly cited reasons for foregoing mental health care.^3,4^ Historically, the costs to patients for equivalent mental health and medical care were not commensurate because mental health and medical conditions were treated in separate health care systems with unique health insurance coverage and payment requirements.^5,6^ These separate systems may have perpetuated another barrier to mental health care, stigma.^7^ Multiple policies enacted in 2008 and 2010 addressed these differences by codifying mental health parity (equal health insurance coverage and/or patient cost-sharing for equivalent mental health and medical services) among the US population with employer-sponsored insurance, Medicaid, and Medicare.^6,8,9,10^ By decreasing cost-sharing for mental health care, parity policies were expected to increase the use of mental health treatment.^6,11,12,13,14,15^

Prior to implementation of the Medicare Improvements for Patients and Providers Act of 2008, beneficiaries were responsible for 50% of cost-sharing for outpatient mental health services compared with only 20% for outpatient medical care.^9^ Under the law, 5% cost-sharing reductions for outpatient mental health services were implemented in 2010, 2012, and 2013. In 2014, a 15% cost-sharing reduction was implemented, bringing cost-sharing for outpatient mental health services to 20% and creating parity with equivalent medical care (Medicare parity).

Two previous studies examined the association of Medicare parity with outpatient mental health service use among lower-income beneficiaries with serious mental illness.^16,17^ Neither study found that the cost-sharing reductions led to increased outpatient mental health visits; however, both studies examined cohorts with serious mental illness, including psychotic disorders (eg, schizophrenia) that may require medication and in-patient services as part of treatment protocols.^18^ As such, this group may be less sensitive to price changes for outpatient mental health services. A third study examined the association of Medicare parity with outpatient office visits with a mental health practitioner and found increased service use among low-income White beneficiaries between 2008 and 2018.^19^

Our objective was to evaluate whether Medicare parity was followed by a change in use of outpatient mental health services among beneficiaries with depression, who represent the majority of Medicare beneficiaries with a mental health condition. In 2019, for example, 93% of beneficiaries who reported having a mental health condition reported having depression specifically.^20^ We examined the association between Medicare parity and outpatient mental health service use and out-of-pocket expenditures. We also considered both the dynamic response to the incremental cost-sharing reductions over time until 20% parity was reached and the broader mental health policy context, including the January 2016 US Preventive Services Task Force (USPSTF) recommendation for routine adult depression screening.

## Methods

### Study Data

This economic evaluation analyzed data from the publicly available Medical Expenditure Panel Survey (MEPS) Household Component files from 2008 to 2019. We did not include a longer time horizon given the impact of the COVID-19 pandemic on both mental health service use and delivery beginning in 2020.^21,22^ The Emory University Institutional Review Board determined the study exempt from review and informed consent as it involved secondary research of publicly available data. The study adheres to the Consolidated Health Economic Evaluation Reporting Standards (CHEERS) and Strengthening the Reporting of Observational Studies in Epidemiology (STROBE) reporting guidelines.

The MEPS is designed to be nationally representative and includes an oversample of subpopulations of particular interest from a health policy perspective.^23^ The MEPS Household Component files include deidentified, self-reported data from household survey participants on demographics (eg, age, sex, race and ethnicity [Asian, Black, Hispanic, White, multiracial, and other]) and health conditions along with confirmatory and supplementary information from their health care practitioners, such as diagnostic codes and payments. Sociodemographic characteristics, including race and ethnicity, were included in the descriptive analysis to provide an overview of the sample and assess changes over time. Professional coders assign *International Classification of Diseases, Ninth Revision* (*ICD-9*) and *International Statistical Classification of Diseases, Tenth Revision* (*ICD-10*) codes to information captured for each reported condition and patient encounter.

### Study Sample

The study sample consisted of beneficiaries aged 65 years or older with depression who had Medicare only or Medicare and supplemental private insurance. We identified MEPS participants with depression in each year of data collection using *ICD-9* codes 296, 300, and 311 (2008-2015) and *ICD-10* codes F32, F33, and F34 (2016-2019). We excluded survey participants who indicated that they had Medicare and other public health insurance as they may already have had access to mental health parity through Medicaid. Additionally, nursing home residents are not included in MEPS data.

### Outcomes

We examined 3 outcomes related to use of outpatient mental health services: (1) mean use (the mean number of outpatient mental health visits per beneficiary per year), (2) proportion of use (the proportion of beneficiaries with ≥1 outpatient mental health visit per year), and (3) intensity of use (the mean use among beneficiaries who had ≥1 outpatient mental health visit per year). We also examined out-of-pocket expenditures for outpatient mental health services. Outpatient mental health services included outpatient or office-based visits with mental health practitioners (psychiatrists, psychologists, and clinical social workers) or primary care physicians, who can screen and identify patients with depression, prescribe antidepressant medication, and/or refer patients for mental health treatment. We used the same methods for identifying and coding outpatient mental health services as previous studies that examined trends in depression treatment using MEPS data.^24,25^

### Statistical Analysis

We used an interrupted time series design and analyzed the years of MEPS data cross sectionally. Given the overlapping implementation of mental health parity policies affecting the US population with employer-sponsored insurance, Medicaid, and Medicare during the study time frame (eFigure in Supplement 1), we specified a single-group model appropriate for examining the effects of health policies.^26,27^ The counterfactual in a single-group interrupted time series is estimated using the preintervention level and trend.^26^ We estimated ordinary least squares (OLS) regression models to examine mean use and proportion of use of outpatient mental health services. For intensity of use and out-of-pocket expenditures, we estimated 2-part regression models and conducted Box-Cox tests and modified Park tests to identify the best link and variance function for each model.^28^ We examined the probability of use and expenditure in part 1 using probit and use and expenditure conditional on any use and expenditure in part 2 using generalized linear models. We adjusted all expenditures for inflation to 2019 US dollars using the Consumer Price Index for Medical Care.^29^ We computed descriptive statistics using MEPS person-level sample weights to generate nationally representative estimates of outcome trends. We used unweighted data to estimate the regression models as a preliminary analysis indicated that the unweighted models were better specified when adjusted for heteroskedasticity as appropriate.^30^ We considered results with *P* < .05 to be statistically significant.

We conducted numerous goodness-of-fit tests to check each model, including Durbin-Watson tests for serial autocorrelation and Breusch-Pagan tests for heteroskedasticity. In addition to checking the specification of each 2-part model, we compared each with a log-transformed OLS model. We also conducted 2 sensitivity analyses for each model. We reran each baseline model with additional change points in 2010, 2012, and 2013 to represent the incremental implementation of Medicare parity. Additionally, we reran each baseline model with a change point for the January 2016 USPSTF recommendation that practitioners screen all adults for depression. Since the study cohort comprised Medicare beneficiaries with depression, the USPSTF recommendation could have had a direct influence on mental health service use among this group by bringing depression screening and subsequent diagnosis and treatment referral into routine care. The data were analyzed from June 2, 2023, to June 17, 2024, using RStudio, version 4.3.1 (R Foundation).

## Results

The analysis included 5831 Medicare beneficiaries aged 65 years or older with depression. Using the MEPS person-level survey weights, this number corresponded to a nationally representative sample of 72 436 656 individuals (eTable 1 in Supplement 1). The majority of the cohort each year was female (64.2%-72.2% vs 27.8%-35.8% male), non-Hispanic White (85.4%-90.2% vs 0.5%-2.1% non-Hispanic Asian, 1.9%-6.1% non-Hispanic Black, 3.4%-5.3% Hispanic, and 1.0%-3.8% non-Hispanic multiracial or non-Hispanic other race), enrolled in the Medicare fee-for-service program (56.7%-64.6%), and resided in the South (29.8%-41.0%) (eTable 2 in Supplement 1). Results from the descriptive analysis indicated that the proportion of beneficiaries with depression who had at least 1 outpatient mental health visit during the year increased from 67.1% in 2008 to 99.8% in 2019 (*P* < .001). Among beneficiaries with at least 1 visit, the proportion with 2 or more visits increased from 60.5% in 2008 to 82.3% in 2019, while those with only 1 visit decreased from 39.5% in 2008 to 17.7% in 2019 (*P* < .001) (eTable 3 in Supplement 1). The mean number of outpatient visits increased from 2.25 (95% CI, 1.79-2.71) in 2008 to 4.90 (95% CI, 4.03-5.77) in 2019 (*P* < .001), and the mean number of visits coded as psychotherapy increased from 0.37 (95% CI, 0.04-0.71) in 2008 to 2.01 (95% CI, 1.18-2.84) in 2019 (*P* < .001). Though mean expenditures for outpatient mental health visits increased over time by payer and overall, the differences were not statistically significant.

**Figure 1.**
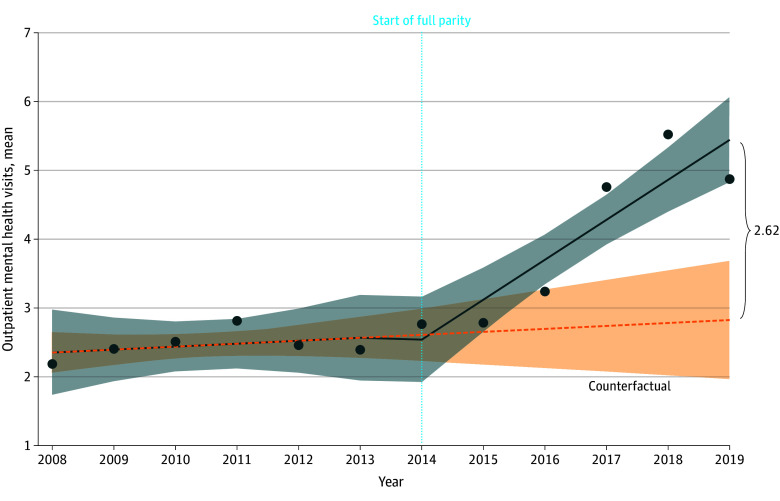
Mean Use of Outpatient Mental Health Services Among Medicare Beneficiaries Aged 65 Years or Older With Depression Interrupted time series model results after implementation of Medicare parity in 2014 (vertical dotted line). Shaded areas indicate the 95% CI.

Regression results indicated that mean use of outpatient mental health services increased significantly after parity by 0.54 visits per year (95% CI, 0.31-0.76 visits per year; *P* < .001) among Medicare beneficiaries with depression (Table 1). This result translates to a relative difference in 2019 of 2.62 visits compared with the counterfactual (Figure 1). Proportion of use increased after parity by 6.61% per year (95% CI, 2.23%-10.99% per year; *P* = .02). Intensity of use decreased at parity by a factor of 0.90 (95% CI, 0.82-1.00; *P* = .04) and increased after parity by a multiple of 1.07 per year (95% CI, 1.04-1.10 per year; *P* < .001). Of note, the 2-part model examining the association of parity with intensity of use failed the Pregibon link goodness-of-fit test, so results are reported for the log-transformed OLS model.

**Table 1.** Medicare Parity and Outpatient Mental Health Service Use and Out-of-Pocket Expenditures Among Beneficiaries Aged 65 Years or Older With Depression, 2008-2019

Variable	OLS				Intensity of use^a^				Out-of-pocket expenditure^b^					
	Mean use^c^		Proportion of use^d^		Log-transformed OLS		Exponentiated coefficient		Part 1. probit		Part 2. GLM: inverse gaussian link = log		Mean marginal effect	
	ITS (95% CI)	*P* value	ITS (95% CI)	*P* value	ITS (95% CI)	*P* value	ITS (95% CI)	*P* value	ITS (95% CI)	*P* value	ITS (95% CI)	*P* value	ITS (95% CI)	*P* value
Preparity trend	0.04 (−0.03 to 0.12)	.25	0.21 (−2.89 to 3.30)	.90	0.0005 (−0.02 to 0.02)	.97	1.00 (0.98 to 1.02)	.97	0.01 (−0.03 to 0.04)	.63	−0.14 (−0.28 to −0.01)	.04	−4.70 (−10.36 to 0.97)	.10
Level change at parity	−0.61 (−1.29 to 0.08)	.08	−4.23 (−19.50 to 11.04)	.61	−0.10 (−0.20 to −0.004)	.04	0.90 (0.82 to 1.00)	.04	−0.14 (−0.30 to 0.02)	.09	0.46 (−0.22 to 1.14)	.19	8.75 (18.09 to 35.60)	.52
Postparity trend	0.54 (0.31 to 0.76)	<.001	6.61 (2.23 to 10.99)	.02	0.07 (0.04 to 0.09)	<.001	1.07 (1.04 to 1.09)	<.001	0.04 (−0.01 to 0.09)	.11	0.27 (0.06 to 0.48)	.01	12.25 (2.42 to 22.08)	.02
Intercept	2.31 (2.05 to 2.57)	<.001	68.91 (56.86 to 80.97)	<.001	0.84 (0.75 to 0.92)	<.001	2.32 (2.12 to 2.51)	<.001	−1.17 (−1.30 to −1.03)	<.001	5.78 (5.19 to 6.38)	<.001	NA	NA
Observations	12	NA	12	NA	4580	NA	NA	NA	5831	NA	771	NA	NA	NA
*R* ^2^	0.894	NA	0.853	NA	0.015	NA	NA	NA	NA	NA	NA	NA	NA	NA
Adjusted *R*^2^	0.855	NA	0.798	NA	0.015	NA	NA	NA	NA	NA	NA	NA	NA	NA
Residual SE	0.438 (*df* = 8)	NA	6.607 (*df* = 8)	NA	0.848 (*df* = 4576)	NA	NA	NA	NA	NA	NA	NA	NA	NA
*F* statistic	22.535 (*df* = 3, 8)	NA	15.529 (*df* = 3, 8)	NA	23.551 (*df* = 3, 4576)	NA	NA	NA	NA	NA	NA	NA	NA	NA
Log likelihood	NA	NA	NA	NA	NA	NA	NA	NA	−2273.078	NA	−4682.762	NA	NA	NA
AIC	NA	NA	NA	NA	NA	NA	NA	NA	4554.157	NA	9373.524	NA	NA	NA

Abbreviations: AIC, Akaike information criterion; GLM, generalized linear model; ITS, interrupted time series; NA, not applicable; OLS, ordinary least squares.

^a^ Defined as mean use among Medicare beneficiaries with depression who had at least 1 outpatient mental health visit per year.

^b^ Defined as expenditures paid by Medicare beneficiaries with depression for outpatient mental health services.

^c^ Defined as the mean number of outpatient mental health visits per year among Medicare beneficiaries with depression.

^d^ Defined as the proportion of Medicare beneficiaries with depression with at least 1 outpatient mental health visit per year.

The trend in out-of-pocket expenditures for outpatient mental health services among beneficiaries with any such expenditure was significantly decreasing before parity by a factor of 0.87 (95% CI, 0.76-0.99; *P* = .04) (Table 1). Of note, descriptions of the 2-part model results reflect exponentiated coefficients given the use of a log link in the model specification. After parity, out-of-pocket expenditures among beneficiaries with any such expenditure increased by a multiple of 1.31 (95% CI, 1.06-1.62; *P* = .01). The mean marginal effects (or combined effects of the 2-part model) indicated that mean out-of-pocket expenditures for outpatient mental health visits increased by approximately $12.25 per year (95% CI, $2.42-$22.08 per year; *P* = .02) after parity.

### Sensitivity Analysis

**Figure 2.**
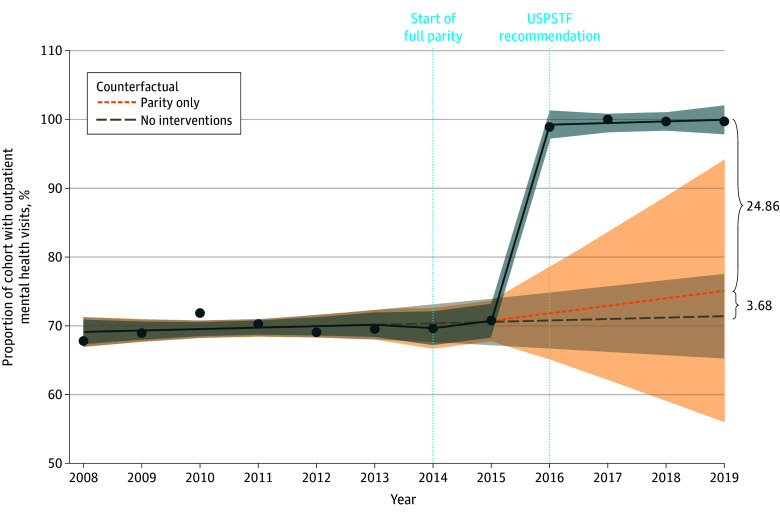
Proportion of Use of Outpatient Mental Health Services Among Medicare Beneficiaries Aged 65 Years or Older With Depression (US Preventive Services Task Force [USPSTF] Model) Sensitivity analysis model with change points for Medicare parity in 2014 and the USPSTF recommendation in 2016 (vertical dotted lines). Shaded areas indicate 95% CIs.

Results from the sensitivity analyses modeling the incremental implementation of parity were similar to results from the baseline models (eTable 4 in Supplement 1). Results from sensitivity analyses accounting for issuance of the USPSTF recommendation (USPSTF model), however, differed from the baseline models (Table 2). In the USPSTF models, changes in mean use, proportion of use, and intensity of use of outpatient mental health services were not significantly associated with cost-sharing parity; rather, statistical significance shifted to issuance of the depression screening guidelines. The trend in mean use increased significantly after the USPSTF recommendation by 0.54 visits per year (95% CI, 0.06-1.02 per year; *P* = .03). Proportion of use increased at the USPSTF recommendation by a level change of 28.26% (95% CI, 24.33%-32.19%; *P* < .001) (Figure 2). The mean marginal effects indicated that intensity of use increased by approximately 0.99 visits (95% CI, 0.44-1.55 visits; *P* < .001) at the time of the USPSTF recommendation and by 0.58 visits per year (95% CI, 0.22-0.93 visits per year; *P* = .001) after it. The USPSTF model examining mean out-of-pocket expenditures produced no significant results associated with either Medicare parity or the USPSTF recommendation (eTable 5 in Supplement 1).

**Table 2.** Sensitivity Analysis Modeling the Association of Medicare Parity and the USPSTF Recommendation on Outpatient Mental Health Service Use Among Beneficiaries Aged 65 Years or Older With Depression, 2008-2019

Variable	OLS				Intensity of use^a^					
	Mean use^b^		Proportion of use^c^		Part 1. probit		Part 2. GLM: Poisson link = log		Mean marginal effects	
	ITS (95% CI)	*P* value	ITS (95% CI)	*P* value	ITS (95% CI)	*P* value	ITS (95% CI)	*P* value	ITS (95% CI)	*P* value
Preparity trend	0.04 (−0.03 to 0.12)	.25	0.21 (−0.39 to 0.80)	.52	0.01 (−0.02 to 0.03)	.72	0.01 (−0.00 to 0.03)	.07	0.05 (−0.01 to 0.10)	.08
Level change at parity	0.17 (−0.08 to 0.42)	.20	−1.59 (−7.43 to 4.24)	.62	−0.04 (−0.29 to 0.21)	.74	0.09 (−0.03 to 0.20)	.13	0.24 (−0.19 to 0.67)	.27
Postparity trend	−0.02 (−0.09 to 0.06)	.68	0.88 (−2.68 to 4.44)	.65	0.03 (−0.12 to 0.18)	.74	−0.02 (−0.09 to 0.05)	.61	−0.03 (−0.29 to 0.22)	.80
Level change at USPSTF	0.39 (−0.83 to 1.62)	.54	28.26 (24.33 to 32.19)	<.001	1.64 (1.08 to 2.20)	<.001	−0.16 (−0.23 to −0.09)	<.001	0.99 (0.44 to 1.55)	<.001
Post-USPSTF trend	0.54 (0.06 to 1.02)	.03	−0.85 (−4.53 to 2.83)	.67	0.18 (−0.11 to 0.48)	.23	0.13 (0.06 to 0.20)	<.001	0.58 (0.22 to 0.93)	.001
Intercept	2.31 (2.05 to 2.57)	<.001	68.91 (66.60 to 71.22)	<.001	0.50 (0.38 to 0.61)	<.001	1.22 (1.16 to 1.27)	<.001	NA	NA
Observations	12	NA	12	NA	5831	NA	4580	NA	NA	NA
*R* ^2^	0.905	NA	0.996	NA	NA	NA	NA	NA	NA	NA
Adjusted *R*^2^	0.827	NA	0.993	NA	NA	NA	NA	NA	NA	NA
Residual SE	0.478 (*df* = 6)	NA	1.267 (*df* = 6)	NA	NA	NA	NA	NA	NA	NA
*F* statistic	11.490 (*df* = 5, 6)	NA	295.529 (*df* = 5, 6)	NA	NA	NA	NA	NA	NA	NA
Log likelihood	NA	NA	NA	NA	−2567.157	NA	−17 363.810	NA	NA	NA
AIC	NA	NA	NA	NA	5146.314	NA	34 739.620	NA	NA	NA

Abbreviations: AIC, Akaike information criterion; GLM, generalized linear model; ITS, interrupted time series; NA, not applicable; OLS, ordinary least squares; USPSTF, US Preventive Services Task Force.

^a^ Defined as mean use among Medicare beneficiaries with depression who had at least 1 outpatient mental health visit per year.

^b^ Defined as the mean number of outpatient mental health visits per year among Medicare beneficiaries with depression.

^c^ Defined as the proportion of Medicare beneficiaries with depression with at least 1 outpatient mental health visit per year.

## Discussion

In this economic evaluation, we examined the dynamic response to the implementation of Medicare parity among beneficiaries with depression within the broader mental health policy landscape. Baseline model results indicated that mean use, proportion of use, and intensity of outpatient mental health service use significantly increased after Medicare parity was implemented. However, sensitivity analyses with the USPSTF recommendation to screen all adults for depression indicated that subsequent changes in clinician practices may have played an important role as coefficients associated with Medicare parity were no longer statistically significant in these models. Since Medicare parity was fully implemented in 2014, results for the trends in outpatient mental health service use after the 2016 USPSTF recommendation may have reflected the influence of both parity cost-sharing reductions and implementation of depression screening. From a policy standpoint, the combination of policies appears more effective than cost-sharing reductions alone. We posit that this apparent synergy is because the two policies were both complementary, as each addressed different barriers to mental health services, and mutually reinforcing, since both may have reduced stigma.

Cost is one of the most commonly cited reasons for foregoing mental health care, and high cost-sharing prior to parity may have contributed to unmet need among Medicare beneficiaries. Medicare parity thus addressed an important barrier to outpatient mental health services by reducing the cost-sharing requirement for beneficiaries from 50% to 20%. By addressing differences in cost-sharing, parity also may have reduced stigma associated with mental health conditions and care seeking.^7^ Medicare parity did not, however, address other barriers to care, including lack of a depression diagnosis or knowledge about depression treatment options and Medicare’s mental health benefits.

By promoting better detection, the USPSTF recommendation may have addressed barriers related to identification and diagnosis of depression. By normalizing adult depression screening as routine care, it also may have reduced stigma around the condition and influenced patient perceptions about the need for and value of treatment. Since the USPSTF recommended screening for depression “with adequate systems in place to ensure accurate diagnosis, effective treatment, and appropriate follow-up,”^31^^(p382)^ the recommendation also may have encouraged health professionals to facilitate outpatient mental health service use by sharing information about depression treatment options and referring patients to mental health practitioners.^32^

It is also important to note the chronologic sequence of the care continuum from depression screening to diagnosis, referral, and treatment relative to the timing and effectiveness of each policy in addressing barriers to care. Diagnosis and referral are usually precursors to the pursuit of treatment, and discussion of treatment options may precede beneficiary knowledge about Medicare’s mental health benefits and patient cost-sharing requirements. Thus, Medicare parity is highly synergistic with the USPTF recommendation as it reduces the cost barrier to seeking treatment once the condition is identified, while depression screening helps identify the condition and prompts treatment seeking. This phenomenon may explain why previous studies examining the association between Medicare parity and service use among beneficiaries with serious mental illness until 2015 and 2016 did not find an increase in outpatient mental health visits.^16,17^

Importantly, it is not possible to isolate the contribution of the USPSTF recommendation to the observed increases in use in this study. Results from the USPSTF models and descriptive analysis, however, support the aforementioned theory. In the year the USPSTF recommendation was implemented, there was a significant increase of 28.26% in the proportion of use among Medicare beneficiaries with depression. As a result, the proportion of Medicare beneficiaries with depression who had any outpatient mental health visits during the year increased from 70.5% in 2015 to 99.1% in 2016 and to nearly 100% the following years. While the increase may reflect increased screening only, since depression screening can be coded as a mental health service, the composition of the visits suggested otherwise. Increases in the number of visits coded as psychotherapy (eTable 1 in Supplement 1) and in the proportion of beneficiaries who had 2 or more visits (eTable 3 in Supplement 1) in the years after issuance of the recommendation indicate that outpatient mental health treatment increased, which may have been facilitated by the reduction in cost-sharing achieved through parity.

Although parity reduced cost-sharing for outpatient mental health services from 50% prior to 2010 to 20% in 2014, our study results indicated that mean out-of-pocket expenditures for these services increased by approximately $12.25 per year after parity among Medicare beneficiaries with depression. This finding may reflect the increase in service use after parity. It also may reflect an increase in outpatient visits with mental health practitioners who may charge higher fees, such as psychiatrists or psychologists, as indicated by the more than 5-fold increase in psychotherapy visits from 2008 to 2019 (eTable 1 in Supplement 1). As such, 20% of higher fees may result in higher out-of-pocket expenditures than higher cost-sharing proportions for less expensive care.

More research is needed to examine the underlying causes for why mean out-of-pocket expenditures for outpatient mental health services increased after Medicare parity despite reductions in beneficiary cost-sharing. Additionally, future studies might examine the association between the 2016 USPSTF recommendation for adult depression screening and physician referral and treatment practices, outpatient mental health service use, and expenditures combined with or separate from policies that established mental health parity. Parity, in conjunction with the USPSTF recommendation, was associated with increased use of outpatient mental health services among Medicare beneficiaries with depression. Further research is needed regarding the extent to which beneficiaries with positive screening results seek and receive treatment and whether treatment is effective and sufficient among those who receive it.

### Limitations

This study used an interrupted time series design to estimate changes in outpatient mental health service use and out-of-pocket expenditures after Medicare parity. Though this method is robust and often used to evaluate the effects of health policies, several limitations must be considered when interpreting the results. First, the results may not be generalizable to all Medicare beneficiaries with depression as the study excluded beneficiaries with dual eligibility for Medicaid or who resided in nursing homes. Moreover, since depression was self-reported and *ICD-9* and *ICD-10* diagnostic codes were later assigned by professional coders, the sample may be subject to recall, social desirability, and/or misclassification bias. Additionally, MEPS person-weights were not used in the estimation of regression models, so regression results may not be nationally representative.

Second, the study used a single-group, interrupted time series design. Though use of a control group may have strengthened the conclusions, given the numerous policies, regulations, and recommendations that affected mental health parity in the US during the study time frame among Medicare beneficiaries, Medicaid enrollees, and individuals with employer-sponsored insurance, a valid control group could not be identified.

Third, the study included no measure of policy awareness or practitioner availability. Additionally, the models did not control for individual-level characteristics that may have confounded the results. Such covariates, however, would not confound the time series results unless they both “predicted the outcome and changed in relationship to the time of the intervention.”^26^^(p308)^

## Conclusions

This economic evaluation found that implementation of mental health parity coupled with the USPSTF recommendation to screen all adults for depression was associated with significant increases in mean use, proportion of use, and intensity of use of outpatient mental health visits among Medicare beneficiaries with depression. Policy makers should consider whether and to what extent individual policies are sufficient to effectively address complex and often interrelated barriers to access of needed mental health care or whether a multipronged approach is warranted. They should examine opportunities for synergy between government policies and health care practices to best leverage the role of each in increasing the availability and accessibility of mental health services.

## Notes

Supplement 1. **eTable 1.** Trends in Use of Outpatient Mental Health Services and Costs Among Medicare Beneficiaries Aged 65 Years or Older With Depression, 2008-2019 **eTable 2.** Sociodemographic Characteristics of Medicare Beneficiaries Aged 65 Years or Older With Depression (2008-2019) **eTable 3.** Number of Outpatient Mental Health Visits Among Medicare Beneficiaries Aged 65 Years or Older With Depression and Any Visits (2008-2019) **eTable 4.** Sensitivity Analysis Modeling Associations Between the Incremental Implementation of Medicare Parity and Outpatient Mental Health Service Use Among Beneficiaries Aged 65 Years or Older With Depression (2008-2019) **eTable 5.** Sensitivity Analysis Modeling the Association Between Medicare Parity and the USPSTF Recommendation on Out-of-Pocket Expenditures for Outpatient Mental Health Services Among Beneficiaries Aged 65 Years or Older With Depression (2008-2019) **eFigure.** Timeline of US Federal Mental Health Policies, Rules, and Recommendations (2008-2019)

Supplement 2.
Data Sharing Statement
