# Supplementary material for: Medicare Parity and Outpatient Mental Health Service Use and Costs Among Beneficiaries With Depression
Source: JAMA Netw Open. 2025 May 2;8(5):e258491. doi: 10.1001/jamanetworkopen.2025.8491 (PMC12048850; doi:10.1001/jamanetworkopen.2025.8491)
Supplement: Supplement 1. — eTable 1. Trends in Use of Outpatient Mental Health Services and Costs Among Medicare Beneficiaries Aged 65 Years or Older With Depression, 2008-2019 eTable 2. Sociodemographic Characteristics of Medicare Beneficiaries Aged 65 Years or Older With Depression (2008-2019) eTable 3. Number of Outpatient Mental Health Visits Among Medicare Beneficiaries Aged 65 Years or Older With Depression and Any Visits (2008-2019) eTable 4. Sensitivity Analysis Modeling Associations Between the Incremental Implementation of Medicare Parity and Outpatient Mental Health Service Use Among Beneficiaries Aged 65 Years or Older With Depression (2008-2019) eTable 5. Sensitivity Analysis Modeling the Association Between Medicare Parity and the USPSTF Recommendation on Out-of-Pocket Expenditures for Outpatient Mental Health Services Among Beneficiaries Aged 65 Years or Older With Depression (2008-2019) eFigure. Timeline of US Federal Mental Health Policies, Rules, and Recommendations (2008-2019) [file jamanetwopen-e258491-s001.pdf]

Supplemental Online Content

Tetlow SM, Phillips VL, Hockenberry JM. Medicare parity and outpatient mental health service use and costs among beneficiaries with depression. *JAMA Netw Open*. 2025;8(5):e258491. doi:10.1001/jamanetworkopen.2025.8491

eTable 1. Trends in Use of Outpatient Mental Health Services and Costs Among Medicare Beneficiaries Aged 65 Years or Older With Depression, 2008-2019
eTable 2. Sociodemographic Characteristics of Medicare Beneficiaries Aged 65 Years or Older With Depression (2008-2019)
eTable 3. Number of Outpatient Mental Health Visits Among Medicare Beneficiaries Aged 65 Years or Older With Depression and Any Visits (2008-2019)
eTable 4. Sensitivity Analysis Modeling Associations Between the Incremental Implementation of Medicare Parity and Outpatient Mental Health Service Use Among Beneficiaries Aged 65 Years or Older With Depression (2008-2019)
eTable 5. Sensitivity Analysis Modeling the Association Between Medicare Parity and the USPSTF Recommendation on Out-of-Pocket Expenditures for Outpatient Mental Health Services Among Beneficiaries Aged 65 Years or Older With Depression (2008-2019)
eFigure. Timeline of US Federal Mental Health Policies, Rules, and Recommendations (2008-2019)

This supplemental material has been provided by the authors to give readers additional information about their work.

© 2025 Tetlow SM et al. *JAMA Network Open*.

eTable 1. Trends in Use of Outpatient Mental Health Services and Costs Among Medicare Beneficiaries Aged 65 Years or Older With Depression, 2008-2019

Treatment-related variable	Mean (95% CI)												*P* value
	2008	2009	2010	2011	2012	2013	2014	2015	2016	2017	2018	2019	
Unweighted No. of beneficiaries (N = 5831)	432	465	432	492	534	539	580	646	448	441	403	419	NA
Weighted No. of beneficiaries (N = 72 436 656)	5 465 454	5 324 784	5 485 234	6 150 886	6 757 716	7 300 120	8 211 770	8 370 776	5 805 906	5 137 661	4 209 452	4 216 898	NA
**Outpatient mental health visits**													
Beneficiaries with ≥1 outpatient visit, No. (%)	3 668 865 (67.1)	3 593 329 (67.5)	4 047 343 (73.8)	4 367 536 (71.0)	4 693 284 (69.5)	5 137 584 (70.4)	5 665 762 (69.0)	5 902 850 (70.5)	5 752 231 (99.1)	5 137 661 (100)	4 193 886 (99.6)	4 208 263 (99.8)	<.001
Outpatient visits, No.	2.25 (1.79-2.71)	2.49 (2.01-2.97)	2.65 (2.26-3.05)	2.88 (2.31-3.45)	2.71 (2.05-3.38)	2.55 (2.11-2.99)	2.85 (2.30-3.41)	2.73 (2.19-3.28)	3.28 (2.84-3.73)	4.82 (3.81-5.84)	4.94 (4.19-5.70)	4.90 (4.03-5.77)	<.001
Psychotherapy visits, No.	0.37 (0.04-0.71)	0.45 (0.10-0.80)	0.44 (0.13-0.75)	0.35 (0.18-0.52)	0.40 (0.18-0.61)	0.54 (0.19-0.89)	0.82 (0.37-1.28)	0.72 (0.25-1.19)	0.61 (0.29-0.93)	1.81 (0.90-2.71)	1.63 (0.98-2.27)	2.01 (1.18-2.84)	<.001
**Outpatient mental health expenditures by payment source, $**													
Self-payment	17.32 (6.33-28.31)	42.80 (7.91-77.69)	40.33 (0.63-80.03)	27.07 (10.82-43.31)	18.74 (10.00-27.48)	20.98 (9.33-32.64)	30.13 (10.74-49.52)	39.65 (20.65-58.65)	31.04 (11.16-50.92)	62.13 (2.33-121.93)	73.27 (10.45-136.10)	77.73 (17.30-138.15)	.54
Medicare	65.79 (36.08-95.51)	94.90 (59.05-130.76)	106.93 (55.40-158.46)	174.38 (52.05-296.71)	102.49 (61.34-143.64)	105.88 (59.45-152.31)	155.76 (79.02-232.51)	101.71 (60.55-142.88)	99.90 (42.54-157.27)	339.48 (63.51-615.45)	259.26 (39.35-479.18)	218.11 (124.76-311.45)	.16
Private insurance	43.58 (9.62-77.54)	50.73 (17.18-84.27)	37.22 (15.55-58.90)	107.51 (9.47-205.55)	44.61 (13.97-75.24)	26.04 (2.83-49.26)	20.41 (10.25-30.56)	73.30 (15.51-131.08)	18.90 (7.42-30.38)	72.65 (14.34-130.95)	35.17 (17.66-52.69)	51.56 (33.09-70.02)	.34
Total from all sources	166.21 (94.11-238.32)	215.45 (139.98-290.92)	211.57 (124.40-298.73)	388.45 (178.24-598.67)	261.62 (127.85-395.39)	171.36 (108.99-233.73)	263.38 (157.95-368.81)	246.24 (158.31-334.17)	176.82 (104.78-248.85)	594.10 (205.61-982.58)	417.79 (186.17-649.42)	370.51 (244.88-496.15)	.14

Abbreviation: NA, not applicable.

eTable 2. Sociodemographic Characteristics of Medicare Beneficiaries Aged 65 Years or Older With Depression (2008-2019), N=72,436,656

Variable, n (%)	**2008** N =5,465,454	**2009** N =5,324,784	**2010** N =5,485,234	**2011** N =6,150,886	**2012** N =6,757,716	**2013** N =7,300,120	**2014** N =8,211,770	**2015** N =8,370,776	**2016** N =5,805,906	**2017** N =5,137,661	**2018** N =4,209,452	**2019** N =4,216,898	p-value
**Age group**													0.48
65-69	1,709,429 (31.3)	1,792,145 (33.7)	1,765,130 (32.2)	2,071,674 (33.7)	2,392,301 (35.4)	2,372,193 (32.5)	3,074,255 (37.4)	3,216,210 (38.4)	2,230,601 (38.4)	1,926,533 (37.5)	1,474,501 (35.0)	1,145,302 (27.2)	
70-74	1,246,521 (22.8)	1,188,518 (22.3)	1,352,801 (24.7)	1,420,992 (23.1)	1,514,177 (22.4)	2,036,373 (27.9)	1,897,117 (23.1)	1,849,793 (22.1)	1,253,292 (21.6)	1,224,427 (23.8)	1,152,015 (27.4)	1,243,522 (29.5)	
75-79	1,095,776 (20.0)	935,574 (17.6)	1,055,178 (19.2)	1,221,594 (19.9)	1,205,876 (17.8)	1,432,288 (19.6)	1,441,660 (17.6)	1,420,698 (17.0)	857,530 (14.8)	780,939 (15.2)	670,594 (15.9)	833,321 (19.8)	
80-84	815,105 (14.9)	768,537 (14.4)	689,935 (12.6)	783,062 (12.7)	709,776 (10.5)	662,734 (9.1)	969,419 (11.8)	881,892 (10.5)	840,497 (14.5)	734,275 (14.3)	419,053 (10.0)	535,688 (12.7)	
85+	598,622 (11.0)	640,010 (12.0)	622,191 (11.3)	653,564 (10.6)	935,586 (13.8)	796,531 (10.9)	829,319 (10.1)	1,002,182 (12.0)	623,987 (10.7)	471,487 (9.2)	493,288 (11.7)	459,066 (10.9)	
**Sex**													0.31
Female	3,944,455 (72.2)	3,750,289 (70.4)	3,552,349 (64.8)	4,095,741 (66.6)	4,337,110 (64.2)	4,842,136 (66.3)	5,497,393 (66.9)	5,441,294 (65.0)	3,938,958 (67.8)	3,381,218 (65.8)	2,743,680 (65.2)	2,981,216 (70.7)	
Male	1,520,999 (27.8)	1,574,495 (29.6)	1,932,886 (35.2)	2,055,146 (33.4)	2,420,605 (35.8)	2,457,983 (33.7)	2,714,377 (33.1)	2,929,482 (35.0)	1,866,948 (32.2)	1,756,443 (34.2)	1,465,771 (34.8)	1,235,682 (29.3)	
**Race and ethnicity**													0.18
NH White	4,877,615 (89.2)	4,749,154 (89.2)	4,927,228 (89.8)	5,377,334 (87.4)	5,893,005 (87.2)	6,231,507 (85.4)	7,299,763 (88.9)	7,420,969 (88.7)	5,205,654 (89.7)	4,560,841 (88.8)	3,797,390 (90.2)	3,741,140 (88.7)	
NH Black	226,623 (4.1)	200,609 (3.8)	160,045 (2.9)	247,527 (4.0)	313,640 (4.6)	447,011 (6.1)	324,928 (4.0)	319,737 (3.8)	177,648 (3.1)	155,666 (3.0)	80,385 (1.9)	117,565 (2.8)	
Hispanic	212,644 (3.9)	184,492 (3.5)	246,700 (4.5)	282,822 (4.6)	232,266 (3.4)	302,227 (4.1)	337,647 (4.1)	398,549 (4.8)	262,579 (4.5)	263,190 (5.1)	223,218 (5.3)	209,895 (5.0)	
NH Asian	38,205 (0.7)	32,567 (0.6)	44,931 (0.8)	99,986 (1.6)	63,913 (0.9)	39,214 (0.5)	60,197 (0.7)	69,864 (0.8)	70,970 (1.2)	107,426 (2.1)	59,622 (1.4)	74,503 (1.8)	
NH Other/ Multiple	110,367 (2.0)	157,962 (3.0)	106,330 (1.9)	143,218 (2.3)	254,891 (3.8)	280,161 (3.8)	189,235 (2.3)	161,657 (1.9)	89,055 (1.5)	50,538 (1.0)	48,836 (1.2)	73,796 (1.8)	
**Medicare type**													0.50
Fee For Service	3,309,253 (60.5)	3,183,956 (59.8)	3,489,255 (63.6)	3,844,657 (62.5)	3,834,437 (56.7)	4,599,145 (63.0)	5,226,903 (63.7)	5,062,272 (60.5)	3,752,889 (64.6)	2,924,584 (56.9)	2,583,675 (61.4)	2,428,851 (57.6)	
Managed Care	1,802,670 (33.0)	1,858,927 (34.9)	1,758,917 (32.1)	1,962,396 (31.9)	2,670,818 (39.5)	2,370,166 (32.5)	2,575,464 (31.4)	2,868,572 (34.3)	1,818,300 (31.3)	1,832,705 (35.7)	1,495,213 (35.5)	1,448,231 (34.3)	
Unknown managed care status	353,531 (6.5)	281,901 (5.3)	237,062 (4.3)	343,833 (5.6)	252,460 (3.7)	330,809 (4.5)	409,403 (5.0)	439,932 (5.3)	234,717 (4.0)	380,373 (7.4)	130,564 (3.1)	339,816 (8.1)	
**Education**													<0.001
0-12 Years	987,343 (18.1)	964,111 (18.1)	896,593 (16.3)	924,252 (15.0)	950,480 (14.1)	738,940 (10.1)	840,611 (10.2)	885,348 (10.6)	605,979 (10.4)	454,764 (8.9)	273,814 (6.5)	328,126 (7.8)	
High school diploma or GED	2,922,664 (53.5)	2,966,325 (55.7)	3,029,006 (55.2)	3,415,007 (55.5)	4,153,292 (61.5)	4,729,310 (64.8)	4,714,855 (57.4)	4,607,858 (55.0)	2,703,157 (46.6)	2,476,374 (48.2)	2,103,243 (50.0)	2,071,402 (49.1)	
Bachelors degree	756,533 (13.8)	656,308 (12.3)	664,266 (12.1)	719,931 (11.7)	903,958 (13.4)	900,429 (12.3)	1,320,082 (16.1)	1,388,440 (16.6)	989,699 (17.0)	850,554 (16.6)	687,463 (16.3)	792,561 (18.8)	
Graduate degree	773,988 (14.2)	714,440 (13.4)	872,586 (15.9)	1,059,841 (17.2)	719,916 (10.7)	863,064 (11.8)	1,281,611 (15.6)	1,439,492 (17.2)	1,438,116 (24.8)	1,270,453 (24.7)	1,142,683 (27.1)	1,010,632 (24.0)	
Not Ascertained or Inapplicable	24,926 (0.5)	23,600 (0.4)	22,784 (0.4)	31,855 (0.5)	30,069 (0.4)	68,376 (0.9)	54,611 (0.7)	49,637 (0.6)	68,955 (1.2)	85,517 (1.7)	2,248 (0.1)	14,177 (0.3)	
**U.S. Region**													0.92
Midwest	1,409,763 (25.8)	1,326,453 (24.9)	1,408,098 (25.7)	1,366,190 (22.2)	1,709,796 (25.3)	1,663,651 (22.8)	2,173,008 (26.5)	2,190,094 (26.2)	1,300,477 (22.4)	1,398,007 (27.2)	836,959 (19.9)	895,725 (21.2)	
Northeast	864,592 (15.8)	831,318 (15.6)	852,997 (15.6)	1,165,546 (18.9)	1,225,265 (18.1)	1,255,084 (17.2)	1,303,921 (15.9)	1,496,951 (17.9)	975,335 (16.8)	891,835 (17.4)	681,594 (16.2)	746,638 (17.7)	
South	2,108,418 (38.6)	1,953,426 (36.7)	1,963,031 (35.8)	2,296,939 (37.3)	2,549,259 (37.7)	2,992,574 (41.0)	3,178,007 (38.7)	2,857,985 (34.1)	1,981,405 (34.1)	1,532,682 (29.8)	1,642,199 (39.0)	1,643,373 (39.0)	
West	1,082,681 (19.8)	1,213,588 (22.8)	1,261,109 (23.0)	1,322,212 (21.5)	1,273,396 (18.8)	1,388,810 (19.0)	1,556,834 (19.0)	1,825,745 (21.8)	1,548,689 (26.7)	1,315,137 (25.6)	1,048,700 (24.9)	931,162 (22.1)	

© 2025 Tetlow SM et al. *JAMA Network Open*.

eTable 3. Number of Outpatient Mental Health Visits Among Medicare Beneficiaries Aged 65 Years or Older With Depression and Any Visits (2008-2019)

Treatment-related variable	2008	2009	2010	2011	2012	2013	2014	2015	2016	2017	2018	2019	p-value
Weighted (n=56,368,595)	3,668,865	3,593,329	4,047,343	4,367,536	4,693,284	5,137,585	5,665,762	5,902,850	5,752,232	5,137,661	4,193,886	4,208,263	
**Outpatient mental health visits, n (%)**													<0.001
1 visit	1,449,013 (39.5)	1,275,255 (35.5)	1,225,774 (30.3)	1,586,771 (36.3)	1,735,982 (37.0)	1,700,018 (33.1)	1,759,392 (31.1)	1,914,374 (32.4)	2,147,306 (37.3)	1,637,732 (31.9)	718,928 (17.1)	744,245 (17.7)	
2 or more visits	2,219,852 (60.5)	2,318,074 (64.5)	2,821,569 (69.7)	2,780,766 (63.7)	2,957,302 (63.0)	3,437,566 (66.9)	3,906,370 (68.9)	3,988,476 (67.6)	3,604,926 (62.7)	3,499,930 (68.1)	3,474,958 (82.9)	3,464,018 (82.3)	

© 2025 Tetlow SM et al. *JAMA Network Open*.

eTable 4. Sensitivity Analysis Modeling Associations Between the Incremental Implementation of Medicare Parity and Outpatient Mental Health Service Use Among Beneficiaries Aged 65 Years or Older With Depression (2008-2019)

Interrupted Time Series (95% CI)				
	**Average use**[1] *OLS*	**Proportion of use**[2] *OLS*	**Intensity of use**[3] Part 1 *Probit*	Part 2 *GLM: Poisson Link = log*
Pre-parity trend	0.100 (-0.067, 0.266)	-0.163 (-5.895, 5.569)	0.050 (-0.002, 0.102)	0.031 (0.007, 0.056)
Level change at parity	0.895 (-2.090, 0.300)	-2.713 (-31.260, 25.835)	-1.024 (-1.311, -0.737)	-0.164 (-0.279, -0.050)
Post-parity trend	0.493 (0.277, 0.708)	7.025 (1.257, 12.794)	0.564 (0.486, 0.641)	0.043 (0.019, 0.068)
Intercept	2.203 (1.885, 2.521)	69.423 (54.484, 84.363)	0.426 (0.287, 0.565)	1.179 (1.111, 1.247)
Observations	12	12	5,831	4,580
R2	0.896	0.853	NA	NA
Adjusted R^2	0.857	0.798	NA	NA
Residual Std. Error	0.435 (df = 8)	6.617 (df = 8)	NA	NA
F Statistic	22.919 (df = 3; 8)	15.475 (df = 3; 8)	NA	NA
Log Likelihood	NA	NA	-2,620.359	-17,382.680
Akaike Inf. Crit.	NA	NA	5,248.719	34,773.360

[1] Average use is defined as the average number of outpatient mental health visits per year among Medicare beneficiaries with depression.
[2] Proportion of use is defined as the proportion of Medicare beneficiaries with depression with at least one outpatient mental health visit per year.
[3] Intensity of use is defined as average use among Medicare beneficiaries with depression who had at least one outpatient mental health visit per year.

© 2025 Tetlow SM et al. *JAMA Network Open*.

eTable 5. Sensitivity Analysis Modeling the Association Between Medicare Parity and the USPSTF Recommendation on Out-of-Pocket Expenditures for Outpatient Mental Health Services Among Beneficiaries Aged 65 Years or Older With Depression (2008-2019)

Interrupted Time Series (95% CI)		
	**Part 1**<br>*Probit*	**Part 2**<br>*GLM: inverse gaussian*<br>*Link = log*
Pre-parity trend	0.009 (-0.025, 0.043)	-0.141 (-0.275, -0.006)
Level change at parity	-0.154 (-0.464, 0.156)	0.008 (-1.305, 1.321)
Post-parity trend	0.063 (-0.122, 0.247)	0.574 (-0.265, 1.413)
Level change at USPSTF	-0.127 (-0.346, 0.092)	-0.171 (-1.285, 0.944)
Post-USPSTF trend	0.007 (-0.186, 0.200)	-0.309 (-1.205, 0.588)
Intercept	-1.166 (-1.303, -1.029)	5.783 (5.194, 6.372)
Observations	5,831	771
Log Likelihood	-2,272.413	-4,682.513
Akaike Inf. Crit.	4,556.826	9,377.027

© 2025 Tetlow SM et al. *JAMA Network Open*.

eFigure. Timeline of US Federal Mental Health Policies, Rules, and Recommendations (2008-2019)

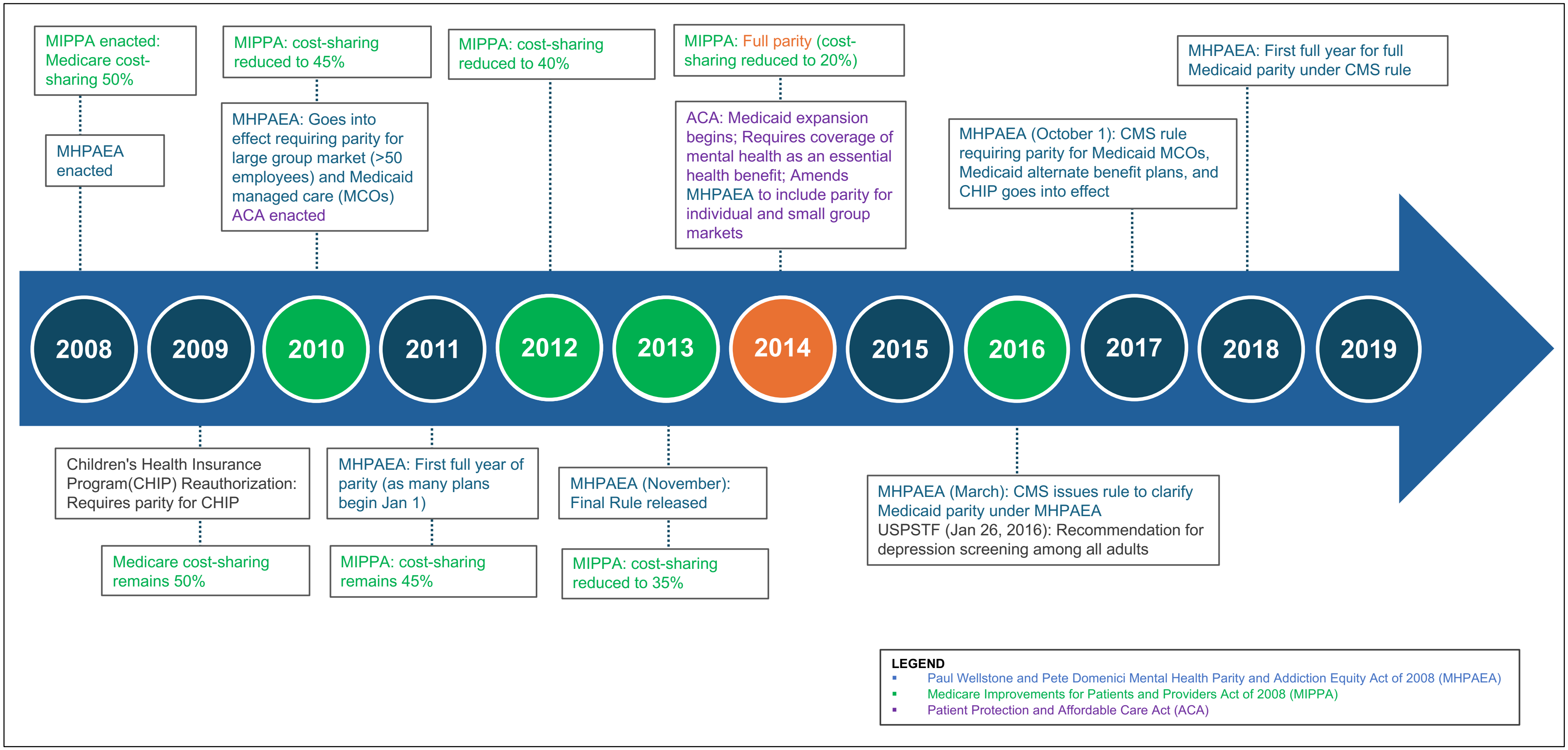

© 2025 Tetlow SM et al. *JAMA Network Open.*
