# Supplementary material for: Medicare Parity and Outpatient Mental Health Service Use and Costs Among Beneficiaries With Depression
Source: JAMA Netw Open. 2025 May 2;8(5):e258491. doi: 10.1001/jamanetworkopen.2025.8491 (PMC12048850; doi:10.1001/jamanetworkopen.2025.8491)
Supplement: Supplement 2. — Data Sharing Statement [file jamanetwopen-e258491-s002.pdf]

Data Sharing Statement

Tetlow. Medicare Parity and Outpatient Mental Health Service Use and Costs Among Beneficiaries With Depression. *JAMA Netw Open*. Published May 02, 2025. doi:10.1001/jamanetworkopen.2025.8491

Data
Data available: No

Additional Information
Explanation for why data not available: The MEPS data used in this study already are publicly available from AHRQ: https://meps.ahrq.gov/mepsweb/survey_comp/household.jsp.
